# Conspiracy beliefs and knowledge about HIV origins among adolescents in Soweto, South Africa

**DOI:** 10.1371/journal.pone.0165087

**Published:** 2017-02-02

**Authors:** Robert Hogg, Busisiwe Nkala, Janan Dietrich, Alexandra Collins, Kalysha Closson, Zishan Cui, Steve Kanters, Jason Chia, Bernard Barhafuma, Alexis Palmer, Angela Kaida, Glenda Gray, Cari Miller

**Affiliations:** 1 Simon Fraser University, Faculty of Health Sciences, Burnaby, British Columbia, Canada; 2 The British Columbia Centre for Excellence in HIV/AIDS, Vancouver, British Columbia, Canada; 3 Perinatal HIV Research Unit, Soweto, Gauteng, South Africa; 4 University of Stellenbosch, Stellenbosch, South Africa; 5 School of Population and Public Health, University of British Columbia, Vancouver, British Columbia, Canada; 6 University of Witswatersrand, Johannesburg, Johannesburg, South Africa; University of New South Wales, AUSTRALIA

## Abstract

**Purpose:**

We examined adolescents’ knowledge regarding the origin of HIV/AIDS and correlates of beliefs surrounding conspiracy theories in Soweto, South Africa. Now, a decade post-AIDS denialism, South Africa has the largest antiretroviral therapy roll-out worldwide. However, conspiracy theories stemming from past AIDS denialism may impact HIV prevention and treatment efforts.

**Methods:**

Study participants were recruited through the Kganya Motsha Adolescent Health Centre and the Perinatal HIV Research Unit’s Botsha Bophelo Adolescent Health Study (BBAHS). Adolescents were eligible to participate if aged 14–19 years and living in Soweto. We calculated the proportion of adolescents who correctly believed that HIV originated from non-human primates, and used contingency table analysis and logistic regression modeling to describe correlates associated with accurate knowledge and beliefs in conspiracy theories.

**Results:**

Of 830 adolescents, 168 (20.2%) participants correctly identified HIV as originating from chimpanzees and one third (n = 71, 8.6%) believed in a conspiracy theory about the origins of HIV, including that it originated from the US government (2.3%), the pharmaceutical industry (2.2%), a vaccine (2.1%), space (1.5%), and a scientist (0.6%). Participants who were more likely to correctly identify the origin of HIV were older, men, and unemployed. Participants who were men, unemployed or students, and who had a parent or close relative who had died of HIV, were more likely to believe in a conspiracy theory regarding the origins of HIV.

**Conclusions:**

Adolescents living in Soweto did not have high levels of accurate knowledge regarding the origins of HIV/AIDS and conspiracy beliefs were present among a small minority of participants. Accurate knowledge of the origins of HIV and debunking myths are important for improving uptake of HIV prevention tools in this population.

## Introduction

Denialist claims and conspiracy theories regarding the origins of HIV/AIDS–identified as skepticism or rejection of biomedical HIV research and treatments–have a widespread history embedded in socio-cultural, political, and religious contexts. For example, poverty [1], witchcraft [2], racial and ethnic oppression [3–5], and antiretroviral medications [1, 6, 7] have often been cited as the source of HIV/AIDS. Such claims have gained acceptance among a significant sub-set of people, including scientists, physicians, and political leaders, notably within the South African context [3, 8, 9].

South Africa continues to experience the world’s most devastating HIV epidemic [10, 11]. AIDS denialism–particularly under past president Thabo Mbeki [3, 8, 12, 13]–has had devastating consequences for South Africans, including a ‘science war’ (i.e. traditional medicine versus antiretroviral medications) [13] that delayed the roll-out of prevention of mother-to-child transmission (PMTCT) and antiretroviral therapy (ART) [1, 8, 14–16], and spurred the uptake of various non-evidence based “cures” for HIV (e.g. ‘virgin cleansing’) [17]. Nattrass [18] has explored the extent to which Mbeki’s AIDS denialist policies impacted AIDS mortality and morbidity in South Africa, finding that an earlier roll out of PMTCT and ARVs (i.e. 1998 versus 2001) would have averted an additional 171,000 HIV infections and prevented approximately 56,000 AIDS-related deaths between 1999 and 2007 [18]. Additionally, a study by Chigwedere and colleagues [19] obtained similar findings, estimating that 330,000 lives were lost to HIV/AIDS and 35,000 babies were born with HIV in South Africa between 2000 and 2005 due to the delay in PMTCT and political inadequacies for country-wide roll out of ART.

By the end of 2014, after approximately 9 years of ART roll-out, 6.8 million people were living with HIV and 140,000 people had died from AIDS in South Africa [10]. Nearly one in five adults aged 15 to 49 years (18.9%) were living with the disease in the country, and among those aged 15 to 24 years, nearly one in ten were infected with HIV [10]. HIV prevention among adolescents is thus a top priority for non-governmental organizations and public health researchers, as the largest percentage of newly HIV-infected people worldwide are aged 15 to 29 years [20, 21].

Understanding the continued impact of AIDS denialism and conspiracy beliefs on the people of South Africa, notably the next generation of South Africans, is of particular importance. HIV prevention is premised on individuals’ abilities to transform their behaviours (e.g. use of condoms during intercourse) based on accurate and evidence-based information [22, 23]. As such, AIDS denialism and conspiracy theories may create obstacles to HIV prevention and treatment efforts, specifically in the South African context which has an extensive history of suspicion and fear originating from Apartheid discourses and racial divisions [9, 24, 25]. It is thus important to understand young people’s knowledge and beliefs about HIV to better prevent new infections. This study examines the relationships between the knowledge that adolescents living in Soweto, South Africa hold in relation to the origins of HIV and related conspiracy theories, and examines potential underlying predictors of HIV knowledge and conspiracy beliefs.

## Methods

Data presented are from the Botsha Bophelo Adolescent Health Study (BBAHS), a cross-sectional observational study exploring socio-behavioural characteristics, HIV testing patterns, and sexual and reproductive health among adolescents (aged 14–19 years) living in Soweto, South Africa. Soweto, a compilation of urban townships established under the apartheid government [26], has approximately 1.2 million residents; 24.7% of whom are 14 years of age and younger [27]. Such demographics, as well as the historical and political backdrop of Soweto, provide an important context in which to explore adolescents’ understandings of the origins of HIV.

Study participants were recruited through the USAID-funded Kganya Motsha Adolescent Centre (KMAC) and the Perinatal HIV Research Unit’s (PHRUs) BBAHS. Adolescents were sampled from all formal and identified informal townships within Soweto using a targeted sampling strategy to ensure age, gender, and neighborhood targets were met. Participants were eligible if aged 14–19 years, living in one of 41 formal and informal neighbourhoods within Soweto, and able to provide informed consent (aged 18–19 years) or parental/caregiver consent and assent to participate (aged <18 years). Recruitment remained open until a sample size of 830 was achieved. Interview-administered surveys were conducted in English using iPads or desktop computers, and took approximately 45–60 minutes. Participants were given an honorarium of 50 ZAR for transportation and associated participation costs. Ethical approval was obtained from the University of Witwatersrand (South Africa) and Simon Fraser University (Canada) research ethics boards.

### Measures

Our principal outcome variable was the dichotomous measure of correctly answering the origin of HIV; namely, that HIV originated from apes and monkeys. Our secondary outcome variable, believing in a conspiracy theory, was created based on groups of other answers relating to a specific theory. Participants believing HIV originated from the US government, the pharmaceutical industry, a vaccine, space, and a scientist were coded as conspiracy beliefs, while remaining participants were classified as not believing in a conspiracy (yes vs. no).

Our explanatory variables included: socio-demographic variables (i.e. age, gender, sexual orientation, education [having grade 8 or higher], employment); living conditions (i.e. living in a shack, food security, HIV in family); and knowledge of HIV. Knowledge of HIV was based on correctly answering all questions to a previously validated 18-item knowledge questionnaire which includes HIV-specific questions relating to HIV prevention, anal sex, and HIV transmission (Study Cronbach alpha = 0.55) [28].

Summary statistics and contingency tables were used to describe the data. Fisher’s exact tests and chi-squared tests were used to determine bivariate associations between each outcome and the explanatory variables. Multivariable logistic regression was subsequently used to identify independent predictors of each outcome among all participants. Model selection was done by choosing the model that minimized Akaike’s information criteria (AIC). All statistical analyses were conducted in SAS version 9.3 (Cary, North Carolina, USA). Data are presented within categories as frequencies [n (%)] or as median and first to third quartile range (Q1-Q3).

## Results

Surveys were conducted with 830 participants between March 2010 and March 2012. The cohort was comprised of 355 (42.8%) young men with a median age of 17 years (Q1-Q3: 16–18). Most respondents (n = 455, 55.0%), were in or had completed high school.

Table 1 presents frequencies for beliefs on the origins of HIV. Of the 830 eligible participants, 168 (20.2%) correctly identified HIV as originating in non-human primates and 71 (8.6%) participants believed in a conspiracy theory, including HIV originating from the US government (2.3%), the pharmaceutical industry (2.2%), a vaccine (2.1%), space (1.5%), and a scientist (0.6%).

**Table 1:** Conspiracy beliefs among adolescents aged 14 to 19 years in Soweto Africa, by sex.

Question/Variable	Categories	Overall	Men	Women	p-value
Where do you think HIV originated/ came from?	Monkeys/apes/ chimpanzees	168 (20.2%)	95 (26.8%)	73 (15.4%)	<0.001
	Scientists invented HIV	5 (0.6%)	3 (0.9%)	2 (0.4%)	
	Space	12 (1.5%)	9 (2.5%)	3 (0.6%)	
	The U.S. government	19 (2.3%)	13 (3.7%)	6 (1.3%)	
	A vaccine	17 (2.1%)	9 (2.5%)	8 (1.7%)	
	Pharmaceutical industry	18 (2.2%)	10 (2.8%)	8 (1.7%)	
	Unsure	374 (45.1%)	127 (35.8%)	247 (52.0%)	
	Other	217 (26.1%)	89 (25.1%)	128 (26.9%)	
Respondent correctly answered origins	Yes	168 (20.2%)	95 (26.8%)	73 (15.4%)	<0.001
	No	662 (79.8%)	260 (73.2%)	402 (84.6%)	
Respondent believes in conspiracy theory	Yes	71 (8.6%)	44 (12.4%)	27 (5.7%)	0.001
	No	759 (91.5%)	311 (87.6%)	448 (94.3%)	
HIV knowledge questions correctly answered	Continuous: Median (Q1-Q3)	14 (12–15)	13 (11–15)	14 (12–16)	<0.001

In the bivariable analysis exploring associations with correctly identifying the origins of HIV (shown in Table 2), correctly answering the origins of HIV was associated with being a man compared to being a woman (56.6% versus 39.9%, p <0.001), being older in age (18–19 years of age) compared to being younger in age (14–15 years of age) (60.7% versus 46.9%, p = 0.006), and being non-employed compared to being a student (20.4% versus 8.8%, p < 0.001). Additionally, Table 3 highlights that participants who were men, unemployed or students, and had a parent or close relative that had died of HIV were correlated with beliefs in conspiracy theories. Reporting high food insecurity was also significantly associated with believing in a conspiracy theory, however it was not selected in the multivariable model.

**Table 2:** Bivariable analysis comparing adolescents’ beliefs about the origin of HIV and other select variables of interest.

Variable	Values	Knows origins	Does not Know	p-value
Sex	Men	95 (56.6%)	260 (39.3%)	<0.001
	Women	73 (43.5%)	402 (60.7%)	
Age category (years)	< = 15	28 (16.7%)	151 (23.2%)	0.006
	15 to 16	38 (22.6%)	195 (29.9%)	
	17 to 19	104 (60.7%)	306 (46.9%)	
Heterosexual	Yes	141 (93.4%)	545 (92.2%)	0.630
	No	10 (6.6%)	46 (7.8%)	
Some high school education	Yes	97 (57.7%)	358 (54.3%)	0.427
	No	71 (42.3%)	301 (45.7%)	
Employed	Yes	4 (2.5%)	25 (4.0%)	<0.001
	No	33 (20.4%)	55 (8.8%)	
	Student	125 (77.2%)	542 (87.1%)	
Lived in a shack	Yes	25 (14.9%)	98 (14.8%)	0.980
	No	143 (85.1%)	564 (85.2%)	
High food insecurity	Yes	93 (55.7%)	339 (51.4%)	0.318
	No	74 (44.3%)	321 (48.6%)	
Parent or close relative had died of HIV	Yes	65 (38.7%)	234 (35.4%)	0.420
	No or unsure	103 (61.3%)	428 (64.7%)	
Correctly answered all HIV knowledge questions	Yes	2 (1.2%)	8 (1.2%)	1.000
	No	166 (98.8%)	654 (98.8%)	

**Table 3:** Bivariable analysis comparing adolescents’ conspiracy beliefs about the origin of HIV and other select variables of interest.

Variable	Values	Believes in conspiracy	Does not believe	p-value
Sex	Men	44 (62.0%)	241 (40.7%)	<0.001
	Women	27 (38.0%)	351 (59.3%)	
Age category (years)	< = 15	14 (20.6%)	125 (21.3%)	0.375
	15 to 16	23 (33.8%)	153 (26.1%)	
	17 to 19	31 (45.6%)	309 (52.6%)	
Heterosexual	Yes	58 (92.1%)	498 (93.1%)	0.793
	No	5 (7.9%)	37 (6.9%)	
Some high school education	Yes	42 (60.0%)	327 (55.2%)	0.448
	No	28 (40.0%)	265 (44.8%)	
Employed	Yes	6 (10.3%)	15 (2.6%)	0.003
	No	3 (5.2%)	70 (12.2%)	
	Student	49 (84.5%)	488 (85.2%)	
Lived in a shack	Yes	8 (11.3%)	85 (14.4%)	0.479
	No	63 (88.7%)	507 (85.6%)	
High food insecurity	Yes	45 (64.3%)	298 (50.4%)	0.318
	No	25 (35.7%)	293 (49.6%)	
Parent or close relative had died of HIV	Yes	37 (52.1%)	209 (35.3%)	0.006
	No or unsure	34 (47.9%)	383 (64.7%)	
Correctly answered all HIV knowledge questions	Yes	1 (1.4%)	7 (1.2%)	0.498
	No	70 (98.6%)	585 (98.8%)	

Table 4 illustrates to results of the multivariable model. Participants who were older (adjusted Odds Ratio [aOR]: 1.70; 95% Confidence Interval [CI]: 1.05–2.75), men (aOR: 2.08; 95% CI: 1.47–2.96), and non-employed (compared to being students, aOR: 2.05, 95% CI: 1.25–3.37; compared to being employed, aOR: 3.55, 95% CI: 1.11–11.36) were more likely to correctly identify the origins of HIV. Additionally, men (aOR: 2.72; 95% CI: 1.60–4.65), those employed (compared to being a student, aOR: 3.79, 95% CI: 1.37–10.50; compared to being unemployed, aOR: 11.26, 95% CI: 2.46–51.59), and those who had a parent or close relative die of HIV (aOR: 2.17; 95% CI: 1.29–3.67) were more likely to believe in a conspiracy origin.

**Table 4:** Multivariable analysis assessing knowledge and conspiracy beliefs about the origin of HIV among all participants.

Variables	Category	Knows origin of HIV aOR (95% CIs)	Believes in conspiracy aOR (95% CIs)
Sex	Men	**Reference**	**Reference**
	Women	0.48 (0.34–0.68)	0.37 (0.22–0.63)
Age category (years)	< = 15	**Reference**	--
	15 to 16	1.04 (0.61–1.79)	
	17 to 19	1.70 (1.05–2.75)	
Employed	No	**Reference**	**Reference**
	Yes	0.28 (0.09–0.90)	11.26 (2.46–51.59)
	Student	0.49 (0.30–0.80)	2.97 (0.89–9.92)
Parent or close relative had died of HIV	No	**--**	**Reference**
	Yes		2.17 (1.29–3.67)

## Discussion

One in five adolescents correctly identified HIV as originating in monkeys and apes and less than one in ten believed in conspiracy theories about HIV’s origin. Participants who correctly identified the origin of HIV were more likely to be older, men, and unemployed. However, conspiracy beliefs were more likely among men, participants who were unemployed or students, and participants who had a parent or close relative who had died of HIV.

Consistent with previous research on individuals’ understandings of HIV/AIDS, particularly among adolescents in South Africa [29–34], our findings demonstrate that adolescents in Soweto remain poorly informed about HIV despite the profound effect of the HIV epidemic on this generation [29]. This underscores how there has been minimal improvement in fully eliminating denialist beliefs and conspiracy theories on the origins of HIV/AIDS with a knowledge gap remaining despite numerous HIV prevention efforts and interventions in South Africa [9, 35, 36]. In particular, these shortcomings indicate that prevention efforts have inadequately addressed the impact of historical, political, and cultural contexts that shape individuals’ conceptualization of illness and disease and multiple kinds of knowledge [13, 37]. Additionally, our findings suggest that the lack of improvement in HIV knowledge among adolescents in South Africa in recent years may remain too low for widespread improvements in HIV prevention uptake among this age group. However, additional research is needed to better understand the impact of conspiracy theory beliefs on HIV prevention successes and failures.

Our findings highlight that adolescents, regardless of sex, showed fairly low HIV knowledge, with less than 2% of participants (14, Q1-Q3: 12–15) able to correctly answer all HIV knowledge questions. Socio-structural factors (e.g. poverty, unemployment, food insecurity) may impact adolescents’ knowledge of HIV/AIDS, which is in line with previous research [36, 38, 39]. For example, participants facing high levels of food insecurity and poverty may need to seek employment opportunities to meet subsistence needs. As such, the association between employment and being unaware of the correct origins of HIV may be related to fewer learning opportunities for adolescents out of school. Increased peer-educational outreach programmes [40] may be beneficial in addressing HIV conspiracy theories and knowledge, particularly for participants who are no longer students.

This is further concerning for adolescent women who were less likely to have accurate knowledge about the origin of HIV, but who are more likely to be impacted by HIV in South Africa. Previous studies have shown that young, Black South African women acquire HIV from five to seven years earlier than their young male peers due to various socio-economic and cultural factors (e.g. intergenerational sexual partnerships, income inequities) [41]. As such, there is a need for targeted, culturally relevant HIV/AIDS education and prevention efforts, as well as structural interventions to address socio-structural conditions faced by our cohort.

This study has several limitations that should be noted. First, the study sample may not be representative of all adolescents in Soweto, despite targeted sampling of formal and informal townships that considered age, gender, and neighborhood characteristics. Second, due to our study sample and study context, our findings may not be generalizable to adolescents elsewhere in South Africa. Third, the modest honorarium offered for study participation (50 ZAR, approximately $6 CAD) may have yielded an over-representation of individuals in need of financial assistance. Fourth, socially desirable responding and recall bias may have affected some of the study variables. Additionally, participants may have been reticent when discussing HIV or uncertain of HIV origins, which may have impacted responses. However, these may have been limited by the use of interviewers who were close to participants’ ages.

## Conclusions

Our data provide important insights into conspiracy beliefs among Soweto adolescents and their determinants. As noted here, almost one in ten adolescents believed in contradictory conspiracy theories about the origins of HIV, and only one in five adolescents could correctly identify HIV as originating in monkeys and apes. Debunking such myths that may impede HIV prevention and treatment among adolescents in this setting will require increased culturally relevant educational efforts by the South African government and by NGOs that represent them.

## Supporting information

S1 File Botsha Bophelo Adolescent Health Study (BBAHS)Interview-administered questionnaire. (PDF)
