# Supplementary material for: Conspiracy beliefs and knowledge about HIV origins among adolescents in Soweto, South Africa
Source: PLoS One. 2017 Feb 2;12(2):e0165087. doi: 10.1371/journal.pone.0165087 (PMC5289418; doi:10.1371/journal.pone.0165087)
Supplement: S1 File — (PDF) [file pone.0165087.s001.pdf]

Coding/Database Management

Date of the Interview:

DD MM YYYY

Day/Month/Year [ ] / [ ] / [ ]

Participant ID #:

[ ]

Interviewer ID #:

[ ]

Interview Location:

- ◯ Kganya Motsha Clinic
- ◯ School
- ◯ Participant's home
- ◯ Restaurant/Other eating place
- ◯ Other (please specify)

[ ]

Interview mode:

- ◯ Interviewer administered via computer
- ◯ Participant administered via Ipad
- ◯ Interviewer administered via Ipad
- ◯ Participant administered via computer

Start time of the interview:

HH MM AM/PM

Time: [ ] : [ ] [▾]

Section A: Socio-demographic information

What is your sex/gender?

- ◯ Female
- ◯ Male
- ◯ Other (please specify)

What is your date of birth?

	DD		MM		YYYY
Day/Month/Year		/		/	

What township of Soweto do you live in?

How long have you lived in Soweto?

- ◯ Since birth
- ◯ Less than 1 year
- ◯ 2 - 5 years
- ◯ 5 - 10 years
- ◯ Greater than 10 years

What ethnic group do you identify with? Please check only one.

- ◯ Zulu
- ◯ Pedi
- ◯ Tsonga
- ◯ Coloured
- ◯ Sotho
- ◯ Swati
- ◯ Tswana
- ◯ Venda
- ◯ Xhosa
- ◯ Ndebele
- ◯ White
- ◯ Other (please specify)

What is the primary (main) language you speak at home? Please check only one.

- ◯ Afrikaans
- ◯ English
- ◯ IsiNdebele
- ◯ IsXhosa
- ◯ IsiZulu
- ◯ Sepedi
- ◯ Sesotho
- ◯ Setswana
- ◯ SiSwati
- ◯ Tshivenda
- ◯ Xitsonga
- ◯ Other (please specify)

What is the highest standard or grade you have completed at school? Please check only one.

- None
- Sub-standard A/Grade 1
- Sub-standard B/Grade 2
- Standard 1/Grade 3
- Standard 2/Grade 4
- Standard 3/Grade 5
- Standard 4/Grade 6
- Standard 5/Grade 7
- Standard 6/Grade 8
- Standard 7/Grade 9
- Standard 8/Grade 10
- Standard 9/Grade 11
- Standard 10/Grade 12
- Incomplete post-secondary training of any kind (university, college, vocational, etc.)
- Complete post-secondary training of any kind (university, college, vocational, etc.)
- Other (please specify)

Are you currently studying in school? (either secondary of post-secondary)

- Yes
- No

Why are you are not currently studying? Please read the entire list and then check all that apply to you.

- [ ] High school is complete
- [ ] Working
- [ ] School is too far away/have no transportation to get there
- [ ] No money for fees
- [ ] Too much violence at school
- [ ] Responsibilities at home
- [ ] Pregnant or caring for my baby/children
- [ ] Illness
- [ ] Was failing/doing poorly
- [ ] School is boring/uninteresting
- [ ] Caring for sick parents or siblings
- [ ] Caring for other sick relatives
- [ ] No particular reason
- [ ] Financial responsibilities at home
- [ ] Other (please specify)

Are you currently working?

- ○ Full-time employed
- ○ Part-time employed
- ○ Self-employed
- ○ Unemployed
- ○ Student
- ○ Working illegally
- ○ Prefer not to answer

From where/whom do you usually get your spending money?

- ○ Employment
- ○ Mother
- ○ Father
- ○ Other relatives
- ○ Boyfriend/girlfriend
- ○ Other (please specify)

What kind of house do you currently live in? Please read the entire list and check only one.

- ○ Brick house ownded by family
- ○ Flat owned by family
- ○ RDP house
- ○ Shack
- ○ Brick house that the family is renting
- ○ Flat that the family is renting
- ○ Hostel
- ○ No House
- ○ Other (please specify)

What is your main source of drinking water? Please read the entire list and check only one answer.

- ◯ Tap water in home
- ◯ Water tank/carrier
- ◯ Community tap
- ◯ Other (please specify)

For each of the following questions, consider what has happened in the past 30 days. Please answer whether this happened never, rarely (once or twice), sometimes (3 - 10 times), or often (more than 20 times) in the past 30 days.

	Never	Rarely	Sometimes	Often
Did you worry that your household would not have enough food?	○	○	○	○
Were you or any household member not able to eat the kinds of food you preferred because of a lack of resources?	○	○	○	○
Did you or any household member eat just a few kinds of food day after day due to a lack of resources?	○	○	○	○
Did you or any household member eat food that you preferred not to eat because of a lack of resources to obtain other types of food?	○	○	○	○
Did you or any household member eat a smaller meal than you felt you needed because there was not enough food?	○	○	○	○
Did you or any household member eat fewer meals in a day because there was not enough food?	○	○	○	○
Was there ever no food at all in your household because there were not resources to get more?	○	○	○	○
Did you or any household member go to sleep at night hungry because there was not enough food?	○	○	○	○
Did you or any household member go a whole day without eating anything because there was not enough food?	○	○	○	○

How many people currently live in your house NOT including yourself?

(By this we mean children and adults who sleep in the household at least two nights or more every week.)

Number of children aged 19 or younger:

Number of adults (aged 20 years or older):

Total number of people:

Please indicate the number of people in each category listed below who live in your household, NOT including yourself.

	1	2	3	4	5
Father	○	○	○	○	○
Mother	○	○	○	○	○
Step-father	○	○	○	○	○
Step-mother	○	○	○	○	○
Mother's boyfriend	○	○	○	○	○
Father's girlfriend	○	○	○	○	○
Grandfather	○	○	○	○	○
Grandmother	○	○	○	○	○
Aunt	○	○	○	○	○
Uncle	○	○	○	○	○
Older brother(s)	○	○	○	○	○
Older sister(s)	○	○	○	○	○
Younger brother(s)	○	○	○	○	○
Younger sister(s)	○	○	○	○	○
Cousin(s)	○	○	○	○	○
My own son/daughter	○	○	○	○	○
Other relative (please specify who in the comment box below)	○	○	○	○	○
Friend	○	○	○	○	○
Acquaintance	○	○	○	○	○
Other People (please specify who in the comment box below)	○	○	○	○	○

Other relative/people (please specify)

Are there any adults aged 20 years or older living with you currently? (i.e. are you living only by yourself or with siblings or is there at least one person who is aged 20 years or older)?

- ◯ Yes
- ◯ No
- ◯ Unsure
- ◯ Prefer not to answer

Is your mom alive?

- ◯ Yes
- ◯ No
- ◯ Unsure
- ◯ Prefer not to answer

What did your mother die of?

- ◯ AIDS or related complications
- ◯ Diarrhoea
- ◯ Diabetes
- ◯ Suicide
- ◯ Killed by boyfriend / husband
- ◯ Killed by person other then boyfriend / husband
- ◯ Heart Problem / Stroke / High blood pressure
- ◯ Tuberculosis (TB)
- ◯ Traffic accident
- ◯ Unsure
- ◯ Prefer not to answer
- ◯ Other (please specify)

Is your father alive?

- ◯ Yes
- ◯ No
- ◯ Unsure
- ◯ Prefer not to answer

What did your father die of?

- Cancer
- Heart Problems/ High Blood Pressure / Stroke
- Diabetes
- Suicide
- Killed by a person
- Diarrhoea
- AIDS or related complications
- Tuberculosis (TB)
- Traffic accident
- Unsure
- Prefer not to answer
- Other (please specify)

In the past 12 months, has anyone in your household received any of the following social grant sources of income?

	Yes	No	Don't know	Prefer not to answer
Social grant: disability	○	○	○	○
Social grant: old age pension	○	○	○	○
Social grant: child support	○	○	○	○
Other (please specify in the comment box below)	○	○	○	○

Other (please specify)

Are you responsible for the care of others in your family who are currently ill?

- ○ Yes
- ○ No

Who is that? Please check all that apply.

- [ ] Mom who is ill
- [ ] Dad who is ill
- [ ] Grandparent/guarding or other relative who is ill
- [ ] Sibling who is ill
- [ ] Other (please specify)

What is the person(s) you are caring for ill with?

- ○ HIV/AIDS related symptoms
- ○ TB
- ○ Pneumonia
- ○ Diarrhoea
- ○ Cancer
- ○ Heart Problems / Stroke / Blood Pressure
- ○ Diabetes
- ○ Injured by accident
- ○ Depression / Mental illness
- ○ Other illness

Section B: Medical History

We are going to ask you a few questions about your health and where you seek health care. In this section we will ask you about family planning, pregnancy, and some sexual health questions including HIV testing. Please ask us if you have any questions about what we are asking.

Have you gone for medical/health care in the last six months?

- ◯ Yes
- ◯ No
- ◯ Unsure

Where did you go for medical/health care? Please read entire list and check all that apply to you.

- [ ] School clinic
- [ ] Medical clinic
- [ ] Family planning clinic
- [ ] Doctor's office
- [ ] Traditional and/or faith healer
- [ ] Kganya Motsha
- [ ] Homeopath
- [ ] Other (please specify)

What was the reason you required medical or health care in the last 6 months? Please check all that apply.

- [ ] Condom broke
- [ ] Concerned about HIV
- [ ] Concerned about other sexually transmitted infections (STIs). By sexually transmitted infection we mean an infection on the penis/vagina that causes discharge, burning, itching, sores, or blisters.
- [ ] Birth control
- [ ] Abortion
- [ ] Pregnancy test
- [ ] Concerned about Tuberculosis (TB)
- [ ] Flu-like symptoms
- [ ] Depression
- [ ] Suicidal thoughts
- [ ] Physically assaulted
- [ ] Sexually assaulted
- [ ] Traffic accident
- [ ] Injury
- [ ] Other (please specify)

What social and health services would you or people you know like to have that you cannot currently find? Please read the entire list and check all that apply.

- [ ] General health services (flu, etc.)
- [ ] Tuberculosis (TB)
- [ ] Abortion
- [ ] Gynecological services (Pap smears, etc.)
- [ ] Counselling (For sexual abuse, violence, family, etc)
- [ ] Reproductive health services (Birth control, pregnancy, sexually transmitted infections (STIs), etc.)
- [ ] Referral to circumcision
- [ ] Addictions counselling (Drugs, alcohol, etc.)
- [ ] Nothing
- [ ] Other (please specify)

If you need to see a health care provider, where is the first place you would fo for care? Please check only one.

- ◯ Nearest medical clinic
- ◯ Another clinic (not the nearest)
- ◯ Doctor's office
- ◯ Traditional and/or faith healer
- ◯ Kganya Motsha
- ◯ Homeopath
- ◯ Other (please specify)

How did you find out about Kganya Motsha? Please read the entire list and check only one.

- ◯ Doctor
- ◯ Mother
- ◯ Father
- ◯ Other relative
- ◯ Friend
- ◯ School/Teacher
- ◯ Pamphlet
- ◯ Clinic recruiter
- ◯ Radio
- ◯ Research Project
- ◯ Other (please specify)

Do you think Kganya Motsha is a youth friendly environment?

- ◯ Yes
- ◯ No
- ◯ Unsure
- ◯ Not Applicable

Will you come back to Kganya Motsha for health services?

- ◯ Yes
- ◯ No
- ◯ Not Applicable

In the last 6 months have you been hospitalized for any reason?

- ◯ Yes
- ◯ No

If yes, what was the reason(s)? Please check all that apply.

- ☐ Tuberculosis (TB)
- ☐ Pregnancy
- ☐ Motor vehicle accident
- ☐ Accidental injury
- ☐ Intentional injury (by someone else)
- ☐ Rape
- ☐ AIDS related illness
- ☐ Other (please specify)

Have you EVER had sexual intercourse before? Please check the option that also correctly identifies your gender.

- ◯ Yes, I have had sexual intercourse; I am a male.
- ◯ No, I have not had sexual intercourse; I am a male.
- ◯ Yes, I have had sexual intercourse; I am a female.
- ◯ No, I have not had sexual intercourse; I am a female.

What family planning or pregnancy prevention methods do you use? Check all that apply to you.

- ☐ Male condoms
- ☐ Female condoms
- ☐ The pill
- ☐ Injection
- ☐ None
- ☐ Other (please specify)

Have you EVER been pregnant?

- ○ Yes
- ○ No
- ○ Unsure
- ○ Prefer not to answer

Other (please specify)

How many times have you been pregnant?

- 1
- 2
- 3
- 4
- 5
- Other (please specify)

What was the outcome of the MOST RECENT pregnancy?

- Livebirth
- Terminated/Abortion
- Miscarriage
- Stillbirth
- Unsure
- Prefer not to answer

Would you prefer for your sexual partner to be circumcised?

- ◯ Yes
- ◯ No
- ◯ Unsure

What family planning or pregnancy prevention methods do you use? Please check all that apply to you.

- ☐ Male condoms
- ☐ Female condoms
- ☐ Rely on girlfriend(s) to take the pill
- ☐ Rely on girlfriend(s) to take the injection
- ☐ Rely on the fact that I have been circumcised
- ☐ None
- ☐ Other (please specify)

Have you EVER made/got somebody pregnant?

- ○ Yes
- ○ No
- ○ Unsure
- ○ Prefer not to answer

How many times have you made/got somebody pregnant?

- ○ 1
- ○ 2
- ○ 3
- ○ 4
- ○ 5
- ○ Other (please specify)

What was the outcome of the MOST RECENT pregnancy?

- ○ Livebirth
- ○ Terminated/Abortion
- ○ Miscarriage
- ○ Stillbirth
- ○ Unsure
- ○ Prefer not to answer

Are you circumcised?

- Yes
- No
- Unsure

Would you be willing to get circumcised if it were offered to you?

- Yes
- No
- Unsure

Would traditional or medical circumcision be more acceptable to you?

- ◯ Traditional
- ◯ Medical
- ◯ Unsure

Does being circumcised make sex less pleasurable for the man?

- ◯ Yes
- ◯ No
- ◯ Unsure

Does being circumcised make sex less pleasurable for the woman?

- ◯ Yes
- ◯ No
- ◯ Unsure

Do you believe that being circumcised will prevent males from getting HIV from female sex partners?

- ◯ Yes
- ◯ No
- ◯ Unsure

Do you believe that being circumcised will prevent males from transmitting HIV to female sex partners?

- ◯ Yes
- ◯ No
- ◯ Unsure

Do you wish to have (more) children in the future?

- ◯ Yes
- ◯ No
- ◯ Unsure

Why do you wish to have children?

How many (more) children would you like to have?

- ◯ 1
- ◯ 2
- ◯ 3
- ◯ 4
- ◯ 5
- ◯ Other (please specify)

Why do you not want to have any (more) children?

Have you ever had an abortion performed or a pregnancy terminated?

- ◯ Yes
- ◯ No
- ◯ Unsure
- ◯ Not applicable, I am male.

How many times have you ever had an abortion/terminated pregnancy?

- ○ 1
- ○ 2
- ○ 3
- ○ 4
- ○ 5 or more
- ○ Prefer not to answer

Where was the abortion(s) performed? Please check all that apply.

- ☐ Free government sponsored clinic
- ☐ Private, for-pay medical clinic
- ☐ Unregulated, for-profit clinic
- ☐ Unsure
- ☐ Prefer not to answer
- ☐ Other (please specify)

Did you suffer any health complications associated with the abortion(s) or termination (s) of pregnancy?

- ☐ None
- ☐ Death
- ☐ Infection
- ☐ Sterilization
- ☐ Unsure
- ☐ Prefer not to answer
- ☐ Other (please specify)

Have you ever needed to get an abortion/have a pregnancy terminated and been unable to?

- ◯ Yes
- ◯ No
- ◯ Unsure
- ◯ Prefer not to answer
- ◯ Not applicable, I am male.

Have you ever experienced any of the following? Please check all that apply.

- ☐ Unexplained discharge from the penis/vagina
- ☐ Burning of the penis/vagina
- ☐ Itch of the penis/vagina
- ☐ Sores on the penis/vagina
- ☐ Blisters on the penis/vagina
- ☐ None
- ☐ Unsure
- ☐ Prefer not to answer

Have you ever been disgnosed with a sexually transmitted infection (STI)? Please check all that apply to you.

- [ ] Genital discharge
- [ ] Gonorrhea
- [ ] Syphilis
- [ ] Genetal or rectal warts
- [ ] Genital sores or ulcers
- [ ] Geniral or rectal herpes
- [ ] Pubic lice
- [ ] Trichomonas
- [ ] Pelvic inflammatory disease (women only)
- [ ] HPV
- [ ] None
- [ ] Unsure
- [ ] Prefer not to answer
- [ ] Other (please specify)

If you have EVER refused to test for HIV, what was the reason? Please check all that apply to you.

- [ ] Fear of being positive
- [ ] Fear of dying
- [ ] Fear of being rejected
- [ ] Fear that the results will be disclosed/my confidentiality violated
- [ ] Not applicable
- [ ] Other (please specify)

What would make it easier for you and people your age to test for HIV?

Have you EVER been tested for HIV?

- ( ) Yes
- ( ) No
- ( ) Prefer not to answer

When was the last time you were tested for HIV?

- ○ Today
- ○ Less than 6 months ago
- ○ 6 - 12 months ago
- ○ 13 - 18 months ago
- ○ 19 - 24 months ago
- ○ More than 2 years ago

Why did you get tested for HIV? Please read entire list and check all that apply.

- ☐ Boyfriend/girlfriend wanted me to
- ☐ Parents wanted me to
- ☐ I wasn't feeling well
- ☐ Friend(s) had a test
- ☐ Someone I had sex with is HIV-positive
- ☐ Mom/dad/other relative had died of HIV
- ☐ Part of routine antenatal care
- ☐ I was raped
- ☐ Condom broke
- ☐ Had unprotected sex/intercourse
- ☐ Recuiter from Kganya Motsha suggested it
- ☐ I wanted to know my HIV status
- ☐ Other (please specify)

____

What is the main reason you got tested for HIV? Please check only one.

- ◯ Boyfriend/girlfriend wanted me to
- ◯ Parents wanted me to
- ◯ I wasn't feeling well
- ◯ Friend(s) had a test
- ◯ Someone I had sex with is HIV-positive
- ◯ Mom/dad/other relative had died of HIV
- ◯ Part of routine antenatal care
- ◯ I was raped
- ◯ Condom broke
- ◯ Had unprotected sex/intercourse
- ◯ Recuiter from Kganya Motsha suggested it
- ◯ I wanted to know my HIV status
- ◯ Other (please specify)

What were the results of your last HIV test?

- ◯ Postitive
- ◯ Negative
- ◯ Indeterminate
- ◯ I don't know
- ◯ Prefer not to answer this question

When was it that you tested positive?

	DD		MM		YYYY
Day/month/year		/		/	

Do you have a doctor that you see about HIV?

- ◯ Yes
- ◯ No
- ◯ Unsure

Are you eligible for antiretroviral (ARV) therapies?

- ◯ Yes
- ◯ No
- ◯ Unsure

Are you on antiretroviral (ARV) therapies?

- ◯ Yes
- ◯ No
- ◯ Unsure

Please indicated whether you believe the following statements to be "true" or "false".

	True	False
Coughing and sneezing DO NOT spread HIV.	◯	◯
A person can get HIV by sharing a glass of water with someone who has HIV.	◯	◯
Pulling out the penis before a man climaxes/cums keeps a woman from getting HIV during sex.	◯	◯
A woman can get HIV is she has anal sex with a man.	◯	◯
Showering, or washing one's genitals/private parts, after sex keeps a person from getting HIV.	◯	◯
All pregnant women infected with HIV will have babies born with AIDS.	◯	◯
People who have been infected with HIV quickly show serious signs of being infected.	◯	◯
There is a vaccine that can stop adults from getting HIV.	◯	◯
People are likely to get HIV by deep kissing, putting their tongue in their partner's mouth, if their partner has HIV.	◯	◯
A woman cannot get HIV is she has sex during her period.	◯	◯
There is a female condom that can help decrease a woman's chance of getting HIV.	◯	◯
A natural skin condom works better against HIV than does a latex condom.	◯	◯
A person will NOT get HIV is she or he is taking antibiotics.	◯	◯
Having sex with more than one partner can increase a person's chance of being infected with HIV.	◯	◯
Taking a test for HIV one week after having sex will tell a person if she or he has HIV.	◯	◯
A person can get HIV by	◯	◯

sitting in a hot tub or a swimming pool with a person who has HIV.	○	○
A person can get HIV from oral sex.	○	○
Using Vaseline or baby oil with condoms lowers the chance of getting HIV.	○	○

Where do you think HIV originated/came from? Please check only one answer.

- ◯ Monkeys/Chimpanzees
- ◯ Space
- ◯ The U.S. Government
- ◯ God
- ◯ One man
- ◯ One woman
- ◯ A vaccine
- ◯ From the pharmaceutical industry
- ◯ Unsure
- ◯ Other (please specify)

Section C: Relationships and Sexual Behaviours

We are going to ask you some questions about boyfriends and/or girlfriends and sexual relationships that you may or may not be involved in. We want to stress that any information you share is absolutely confidential. Please also be assured that we will not judge you or any information you share with us. The number of sexual partners people have varies from person to person; there is no right or wrong number. For some young people, it is normal to have sexual partners or girlfriends/boyfriends who are the same sex. Also, while some young people may not be having sexual intercourse, some may be having oral sex. We know these are difficult issues to discuss but would appreciate it if you could tell us now about any types of sexual behaviour you may be engaged in. If you feel you can’t answer honestly please skip to the next question by checking the "Prefer not to answer" option.

How do you identify your sexual orientation?

- ◯ Straight
- ◯ Gay or Lesbian
- ◯ Bi-sexual
- ◯ Undecided/Don't Know
- ◯ Prefer not to answer

Have you had a boyfriend/girlfriend in the past 6 months?

- ◯ Yes
- ◯ No
- ◯ Unsure
- ◯ Prefer not to answer

How many girlfriends/boyfriends have you had in the past 6 months?

- ◯ One
- ◯ Two
- ◯ Three or more
- ◯ Unsure
- ◯ Prefer not to answer

How many boyfriends/girlfriends do you currently have?

- ◯ None
- ◯ One
- ◯ Two
- ◯ Three or more
- ◯ Can't remember
- ◯ Prefer not to answer

In the past 6 months, how old were your boyfriends/girlfriends?

1.	
2.	
3.	
4.	
5.	
6.	
7.	
8.	
9.	
10.	

About how old do you think most of your friends were the first time they had sexual intercourse (penis to vagina or anus)? Please check only one.

- ◯ 10 years or younger
- ◯ 11 - 12 years old
- ◯ 13 - 14 years old
- ◯ 15 - 16 years old
- ◯ 17 - 19 years old

Do you think young people begin to have consensual oral sex (mouth to penis or vagina) before they start having consensual vaginal sex? By consensual we mean you wanted and agreed to have sex.

- ◯ Yes
- ◯ No
- ◯ Unsure

How old do you think most of your friends were the first time they had consensual oral sex? By consensual we mean they wanted and agreed to have oral sex.

- ◯ 10 years old or younger
- ◯ 11 - 12 years old
- ◯ 13 - 14 years old
- ◯ 15 - 16 years old
- ◯ 17 - 19 years old

Do you think a person is at risk for HIV when having oral sex?

- ◯ Yes
- ◯ No
- ◯ Unsure

Have you ever had consensual oral sex before? By consensual we mean you wanted and agreed to have oral sex.

- ◯ Yes
- ◯ No
- ◯ Unsure
- ◯ Prefer not to answer

How old were you the first time you had consensual oral sex?

- 10 years or younger
- 11-12 years old
- 13-14 years old
- 15-16 years old
- 17-18 years old
- 19 years old
- Prefer not to answer

How many people have you ever had consensual oral sex with?

- 1
- 2-4
- 5-7
- 8-10
- 11-15
- 16-20
- 21 or more
- Prefer not to answer

Have you ever had consensual vaginal sex (penis to vagina)? By consensual we mean you wanted and agreed to have vaginal sex.

- ◯ Yes
- ◯ No
- ◯ Unsure
- ◯ Prefer not to answer

How old were you were you the first time you had consensual vaginal sex?

- ◯ 10 years old or younger
- ◯ 11-12 years old
- ◯ 13-14 years old
- ◯ 15-16 years old
- ◯ 17-18 years old
- ◯ 19 years old
- ◯ Prefer not to answer

About how many people have you ever had consensual vaginal sex with in your lifetime?

- ◯ 1
- ◯ 2-4
- ◯ 5-7
- ◯ 8-10
- ◯ 11-15
- ◯ 16-20
- ◯ 21 or more
- ◯ Prefer not to answer

How often did you use a condom when having consensual vaginal sex in your lifetime? By using a condom we mean you used it from start to finish of one sexual act.

- ◯ Always
- ◯ Sometimes
- ◯ Never
- ◯ Unsure
- ◯ Prefer not to answer

Have you ever had consensual anal sex (penis in anus) in your lifetime? By consensual we mean you wanted and agreed to have anal sexual intercourse.

- ◯ Yes
- ◯ No
- ◯ Unsure
- ◯ Prefer not to answer

How old were you the first time you had consensual anal sex?

- 10 years or younger
- 11-12 years old
- 13-14 years old
- 15-16 years old
- 17-18 years old
- 19 years old
- Prefer not to answer

How many people have you ever had consensual anal sex with?

- 1
- 2-4
- 5-7
- 8-10
- 11-15
- 16-20
- 21 or more
- Prefer not to answer

How often did you use condoms when having consensual anal sex in your lifetime? By using a condom we mean you used it from start to finish of one sexual act.

- Always
- Sometimes
- Never
- Unsure
- Prefer not to answer

This section is for people who have had sex with WOMEN in the past 6 months...

We understand that questions about sex are hard to answer but please answer honestly and if you feel you cannot, there is a "Prefer not to answer" option. We appreciate you providing this information and assure you it will be kept confidential.

Have you had any kind of sexual intercourse with a WOMAN in the past 6 months?

- ○ Yes
- ○ No
- ○ Unsure
- ○ Prefer not to answer

How many women have you had consensual vaginal sex with in the past 6 months? By vaginal we mean penis in vagina, and by consensual we mean you wanted and agree to have sex.

- ◯ 0
- ◯ 1
- ◯ 2-4
- ◯ 5-7
- ◯ 8-10
- ◯ 11-15
- ◯ 16-20
- ◯ 21 or more
- ◯ Prefer not to answer

When you had vaginal sex with women in the past six months, how often did you use condoms? By using a condom we mean you used it from start to finish of one sexual act.

- ◯ Always
- ◯ Sometimes
- ◯ Never
- ◯ Unsure
- ◯ Prefer not to answer

What were the ages of the women you had sex with in the past 6 months?

1.
2.
3.
4.
5.
6.
7.
8.
9.
10.

Have you had consensual anal sex with a woman in the past 6 months? By anal sex we mean penis in the anus, and by consensual we mean you wanted and agreed to have sex.

- ◯ Yes
- ◯ No
- ◯ Prefer not to answer

How many women have you had consensual anal sex with in the past 6 months?

- 1
- 2-4
- 5-7
- 8-10
- 11-15
- 16-20
- 21 or more
- Prefer not to answer

How often did you use a condom when having anal sex with women in the past 6 months? By using a condom we mean you used it from start to finish of one sexual act.

- Always
- Sometimes
- Never
- Unsure
- Prefer not to answer

Are you less likely to use a condom when having sexual intercourse with women who are younger or older than you? By using a condom we mean you used it from start to finish of one sexual act.

- ◯ Younger
- ◯ Older
- ◯ No difference
- ◯ Prefer not to answer

In the past 6 months, have you ever found it difficult to get condoms when you need or want them?

- ◯ Yes
- ◯ No
- ◯ Sometimes
- ◯ Prefer not to answer

Why have you found it difficult to get condoms? Please read the entire list and check all that apply.

- [ ] Too expensive
- [ ] Hard to find/too far away
- [ ] I'm embarassed to buy them
- [ ] I'm embarrassed to ask for the free condoms
- [ ] I'm embarassed to pick up condoms from the free dispenser
- [ ] Prefer not to answer
- [ ] Other (please specify)

In the past 6 months, has your sexual partner(s) ever refused to wear a condom or would not permit you to wear a condom when you asked?

- ◯ Yes
- ◯ No
- ◯ Unsure
- ◯ Prefer not to answer

The last time you had sex with a woman, what was the main reason(s)? Please read the entire list and check all that apply.

- ☐ Forced/pressured by partner
- ☐ For food, shelter, clothes, or other goods
- ☐ In love
- ☐ Wanted to feel loved/protected
- ☐ Boredom
- ☐ For pleasure/I like having sex/It's fun
- ☐ Friends were/Everyone was/Did not want to be left out
- ☐ Prefer not to answer
- ☐ Other (please specify)

The last time you had penetrative sexual intercourse (penis in vagina or anus) with a woman, was a condom used? By using a condom we mean you used it from start to finish of one sexual act.

- ◯ Yes
- ◯ No
- ◯ Unsure
- ◯ Prefer not to answer

Why didn't you use a condom?

- [ ] Condoms were not available at the time
- [ ] Asking to use a condom makes your partner thinks she is not trusted
- [ ] I would be embarrassed to use a condom
- [ ] Sex might be spoiled if my partner and I talked about using a condom
- [ ] It is hard to ask to use a condom if my partner and I are already using the pill
- [ ] My partner would be angry if I asked to use a condom
- [ ] It is hard to talk about ways not to get AIDS and other sexually transmitted diseases
- [ ] Sex would be less exciting if condoms were used
- [ ] Sex is less romantic when using condoms
- [ ] Condoms make sex feel less good
- [ ] Prefer not to answer
- [ ] Other (please specify)

Why did you use a condom?

- [ ] Condoms are a good way to stop AIDS and other sexually transmitted infections/diseases (STIs)
- [ ] Condoms are safe to use
- [ ] Using condoms is the responsible thing to do
- [ ] By using condoms, my partner and I are less likely to get AIDS or other STIs
- [ ] Using a condom shows you care about yourself and your partner
- [ ] If I used condoms my partner would respect me
- [ ] To prevent pregnancy
- [ ] Prefer not to answer
- [ ] Other (please specify)

In your current/most recent sexual relationship, would you say that your girlfriend has more control over whether or not you used condoms than you did?

- ◯ Always
- ◯ Sometimes
- ◯ Never
- ◯ Unsure
- ◯ Prefer not to answer

In your current/most recent sexual relationship, does your girlfriend have more control over whether or not you have sex than you do?

- ◯ Always
- ◯ Sometimes
- ◯ Never
- ◯ Unsure
- ◯ Prefer not to answer

Have you ever had sex with someone in order to get material goods such as money, food, presents, clothes, better grades, or other items?

- ◯ Yes
- ◯ No
- ◯ Unsure
- ◯ Prefer not to answer

How many people in the past 6 months have you had sex with for money, food, or other goods?

- ◯ 1
- ◯ 2-4
- ◯ 5-7
- ◯ 8-10
- ◯ 11-15
- ◯ 16-20
- ◯ 21 or more
- ◯ Prefer not to answer

Who was the last person with whom you had sex for money, food, or other goods?

- ◯ Stranger
- ◯ Friend my age
- ◯ Older friend
- ◯ Teacher
- ◯ Someone I know in the neighborhood/and acquaintance
- ◯ Girlfriend
- ◯ Prefer not to answer
- ◯ Other (please specify)

Have you ever given someone material goods such a money, food, presents, clothes, or other items in order for them to have sex with you?

- ◯ Yes
- ◯ No
- ◯ Unsure
- ◯ Prefer not to answer

This section is for people who have had sex with MEN in the past 6 months.

We understand that questions about sex are hard to answer but please answer honestly and if you feel you cannot, there is a "Prefer not to answer" option. We appreciate you providing this information and assure you it will be kept confidential.

Have you had any kind of sexual intercourse with a MAN in the past 6 months?

- ◯ Yes
- ◯ No
- ◯ Unsure
- ◯ Prefer not to answer

How many men have you had consensual vaginal sex with in the past 6 months? By vaginal we mean penis in vagina, and by consensual we mean you wanted and agreed to have sex.

- ◯ 0
- ◯ 1
- ◯ 2-4
- ◯ 5-7
- ◯ 8-10
- ◯ 11-15
- ◯ 16-20
- ◯ 21 or more
- ◯ Prefer not to answer

When you had vaginal sex with men in the past six months, how often did you use condoms? By using a condom we mean you used it from start to finish of one sexual act.

- ◯ Always
- ◯ Sometimes
- ◯ Never
- ◯ Unsure
- ◯ Prefer not to answer

What were the ages of the men you had sex with in the past 6 months?

1.
2.
3.
4.
5.
6.
7.
8.
9.
10.

Have you had consensual anal sex with men in the past 6 months? By anal sex we mean penis in the anus, and by consensual we mean you wanted and agree to have sex.

- ○ Yes
- ○ No
- ○ Prefer not to answer

How many men have you had consensual anal sex with in the past 6 months?

- ◯ 1
- ◯ 2-4
- ◯ 5-7
- ◯ 8-10
- ◯ 11-15
- ◯ 16-20
- ◯ 21 or more
- ◯ Prefer not to answer

How often did you use a condom when having anal sex with men in the past 6 months? By using a condom we mean you used it from start to finish of one sexual act.

- ◯ Always
- ◯ Sometimes
- ◯ Never
- ◯ Unsure
- ◯ Prefer not to answer

Are you less likely to use a condom when having sexual intercourse with men who are younger or older than you? By using a condom we mean you used it from start to finish of one sexual act.

- ◯ Younger
- ◯ Older
- ◯ No difference
- ◯ Prefer not to answer

In the past 6 months, have you ever found it difficult to get condoms when you need or want them?

- ◯ Yes
- ◯ No
- ◯ Sometimes
- ◯ Prefer not to answer

Why have you found it difficult to get condoms? Please read the entire list and check all that apply.

- ☐ Too expensive
- ☐ Hard to find/too far away
- ☐ I'm embarassed to buy them
- ☐ I'm embarrassed to ask for the free condoms
- ☐ I'm embarassed to pick up condoms from the free dispenser
- ☐ Prefer not to answer
- ☐ Other (please specify)

In the past 6 months, has your sexual partner(s) ever refused to wear a condom or would not permit you to wear a condom when you asked?

- ○ Yes
- ○ No
- ○ Unsure
- ○ Prefer not to answer

The last time you had sex with a man, what was the main reason(s)? Please read the entire list and check all that apply.

- ☐ Forced/pressured by partner
- ☐ For food, shelter, clothes, or other goods
- ☐ In love
- ☐ Wanted to feel loved/protected
- ☐ Boredom
- ☐ For pleasure/I like having sex/It's fun
- ☐ Friends were/Everyone was/Did not want to be left out
- ☐ Prefer not to answer
- ☐ Other (please specify)

The last time you had penetrative sexual intercourse (penis in vagina or anus) with a man, was a condom used? By using a condom we mean you used it from start to finish of one sexual act.

- ○ Yes
- ○ No
- ○ Unsure
- ○ Prefer not to answer

Why didn't you use a condom?

- [ ] Condoms were not available at the time
- [ ] Asking to use a condom makes your partner thinks he is not trusted
- [ ] I would be embarrassed to use a condom
- [ ] Sex might be spoiled if my partner and I talked about using a condom
- [ ] It is hard to ask to use a condom if my partner and I are already using the pill
- [ ] My partner would be angry if I asked to use a condom
- [ ] It is hard to talk about ways not to get AIDS and other sexually transmitted diseases
- [ ] Sex would be less exciting if condoms were used
- [ ] Sex is less romantic when using condoms
- [ ] Condoms make sex feel less good
- [ ] Prefer not to answer
- [ ] Other (please specify)

Why did you use a condom?

- [ ] Condoms are a good way to stop AIDS and other sexually transmitted infections/diseases (STIs)
- [ ] Condoms are safe to use
- [ ] Using condoms is the responsible thing to do
- [ ] By using condoms, my partner and I are less likely to get AIDS or other STIs
- [ ] Using a condom shows you care about yourself and your partner
- [ ] If I used condoms my partner would respect me
- [ ] To prevent pregnancy
- [ ] Prefer not to answer
- [ ] Other (please specify)

In your current/most recent sexual relationship, would you say that your boyfriend has more control over whether or not you used condoms than you did?

- ◯ Always
- ◯ Sometimes
- ◯ Never
- ◯ Unsure
- ◯ Prefer not to answer

In your current/most recent sexual relationship, does your boyfriend have more control over whether or not you have sex than you do?

- ◯ Always
- ◯ Sometimes
- ◯ Never
- ◯ Unsure
- ◯ Prefer not to answer

Have you ever had sex with someone in order to get material goods such as money, food, presents, clothes, better grades, or other items?

- ◯ Yes
- ◯ No
- ◯ Unsure
- ◯ Prefer not to answer

How many people in the past 6 months have you had sex with for money, food, or other goods?

- ◯ 1
- ◯ 2-4
- ◯ 5-7
- ◯ 8-10
- ◯ 11-15
- ◯ 16-20
- ◯ 21 or more
- ◯ Prefer not to answer

Who was the last person with whom you had sex for money, food, or other goods?

- ◯ Stranger
- ◯ Friend my age
- ◯ Older friend
- ◯ Teacher
- ◯ Someone I know in the neighborhood/and acquaintance
- ◯ Boyfriend
- ◯ Prefer not to answer
- ◯ Other (please specify)

Have you ever given someone material goods such a money, food, presents, clothes, or other items in order for them to have sex with you?

- ◯ Yes
- ◯ No
- ◯ Unsure
- ◯ Prefer not to answer

We are now going to ask you about some possible sexual experiences that may have happened to you. We recognize that they might be hard to talk about, and if you do not wish to answer them please check the "Prefer not to answer" option. We know that sex is really hard to talk about, particularly sexual violence, but please understand that your honesty is really important so that we can better respond to the risks for HIV in you community. Please also know that sex and sexual violence are unfortunately very common in your age group and in your community. Therefore, you will not be alone in anything you tell us. If you feel you can't or don't want to answer a question honestly, then just check the "Prefer not to answer" option. Also, if anything you tell us leaves you feeling sad or scared, there is a social worker that is available to sit with you after the interview. The information you share with us today is important and will be used to advocate for services to support the health of adolescents.

Has someone ever forced you to have sexual intercourse with them?

- ◯ Yes
- ◯ No
- ◯ Unsure
- ◯ Prefer not to answer

Who was the person who forced you to have sexual intercourse with them? Please read the entire list and check all that apply.

- ☐ Parent
- ☐ Step-parent
- ☐ Other relative
- ☐ Neighbor
- ☐ Someone from school
- ☐ Gang raped
- ☐ Boyfriend/girlfriend
- ☐ Prefer not to answer
- ☐ Other (please specify)

How many different people have forced you to have sex with them in your lifetime?

How old were you the first time someone forced you to have sexual intercourse with them?

Have you ever forced someone to have sexual intercourse with you?

- ◯ Yes
- ◯ No
- ◯ Unsure
- ◯ Prefer not to answer

How many different people have you forced to have sex with you ever?

How old were you the first time you forced someone to have sexual intercourse with you?

Section D: Alcohol and Drug Use

We would like to ask you some questions about drug and alcohol use. Some of the questions that we ask might be uncomfortable. Please remember that anything you share with us about alcohol or drugs will not be shared with anyone else. If you do not want to answer a question, please check the "Prefer not to answer" option.

Have you had alcohol to drink in the past 6 months?

- ◯ Yes
- ◯ No
- ◯ Unsure
- ◯ Prefer not to answer

How often do you drink alcohol?

- ◯ I've only ever had alcohol one time
- ◯ Once a month or less
- ◯ Once a week or less
- ◯ 2 - 3 times per wekk
- ◯ 4 times per week or more
- ◯ Only on special occasions
- ◯ Prefer not to answer

How many times in the past 6 months have you been drunk?

- ◯ Never
- ◯ Once a month or less
- ◯ Once a week or less
- ◯ 2 - 3 times per week
- ◯ 4 times per week or more
- ◯ Only one time
- ◯ Prefer not to answer

Have you ever felt you should cut down on your drinking?

- Yes
- No
- Prefer not to answer

Have people ever annoyed you by criticizing your drinking?

- Yes
- No
- Prefer not to answer

Have you ever felt bad or guilty about your drinking?

- Yes
- No
- Prefer not to answer

Have you ever had a drink first thing in the morning to steady your nerves or get rid of a hangover?

- Yes
- No
- Prefer not to answer

Do any of your friends drink alcohol on a regular basis?

- Yes
- No
- Unsure
- Prefer not to answer

How often have you smoked or snorted any of the following drugs in the past 6 months?

	Not at all	Once a month or less	Once a week or less	More than once per week	At least daily
Marijuana/Pot/Dagga	○	○	○	○	○
Mandrax/Buttons	○	○	○	○	○
Glue	○	○	○	○	○
Heroin	○	○	○	○	○
Tik	○	○	○	○	○
Cocaine/Coke	○	○	○	○	○
Crack/Rocks	○	○	○	○	○
Ecstasy/Methamphetamines	○	○	○	○	○
Petrol/Benzine	○	○	○	○	○
Prescription pills (not including HIV Antiretrovirals)	○	○	○	○	○
LSD/Acid	○	○	○	○	○
HIV Antiretrovirals	○	○	○	○	○
Hooka pipe	○	○	○	○	○
Chotle	○	○	○	○	○
Other (please specify in the comment box below)	○	○	○	○	○

Other (please specify):

Have you used injection drugs in the past 6 months?

- ○ Yes
- ○ No
- ○ Unsure
- ○ Prefer not to answer

What drugs have you injected? Please list all that apply.

How often do you inject?

- ◯ I've only ever done it once
- ◯ Once a month or less
- ◯ Once a week or less
- ◯ 2 - 3 times per week
- ◯ 4 times per week or more
- ◯ Once per day or more
- ◯ Prefer not to answer

Section E: Depression

For each of the following questions, consider how you have felt or behaved during the past week. Please answer whether the following happened rarely or none of the time (less than one day this past week), some or a little of the time (1 - 2 days this past week), occasionally or a moderate amount of the time (3 - 4 days this past week), or most or all of the time (5 - 7 days this past week).

	Rarely or none of the time (less than one day this past week)	Some or a little of the time (1 - 2 days this past week)	Occasionally or a moderate amount of the time (3 - 4 days this past week)	Most or all of the time (5 - 7 days this past week)
I was bothered by things that don't usually bother me.	◯	◯	◯	◯
I did not feel like eating; my appetite was poor.	◯	◯	◯	◯
I felt that I could not shake off the blues even with the help of my family or friends.	◯	◯	◯	◯
I felt that I was just as good as other people.	◯	◯	◯	◯
I had trouble keeping my mind on what I was doing.	◯	◯	◯	◯
I felt depressed.	◯	◯	◯	◯
I felt everything I did was an effort.	◯	◯	◯	◯
I felt hopeful about the future.	◯	◯	◯	◯
I thought my life had been a failure.	◯	◯	◯	◯
I felt fearful.	◯	◯	◯	◯
My sleep was restless.	◯	◯	◯	◯
I was happy.	◯	◯	◯	◯
I talked less than usual.	◯	◯	◯	◯
I felt lonely.	◯	◯	◯	◯
People were unfriendly.	◯	◯	◯	◯
I enjoyed life.	◯	◯	◯	◯
I had crying spells.	◯	◯	◯	◯
I felt sad.	◯	◯	◯	◯
I felt that people disliked me.	◯	◯	◯	◯
I could not get "going".	◯	◯	◯	◯

Section F: Traumatic Events

This is a survey of events and things that might have happened to you while you were growing up. Please answer the questions to the best of your ability. There are no right or wrong answers.

Have you ever experienced any of the following?

	Yes	No
Been separated from your mother or the other person who looks after you for more that three months at a time (for example, lived with another relative or in foster care)?	○	○
Have your parents split up or separate?	○	○
Parents argued frequently or more than usual?	○	○
Changed schools (not because of graduation) or moved to a new home?	○	○
Have your parent or guardian lose their job?	○	○
Lost your home or had no home?	○	○
Has a family member or someone close to you who had HIV or AIDS?	○	○
Has a family member or someone close to you who died of HIV or AIDS?	○	○
Has a family member or someone close to you who died?	○	○
Found out that a family member or someone close to you was very sick or had a bad injury?	○	○
Experienced discrimination based on your race or ethnicity?	○	○
Your family struggled with money, that is, struggled to make ends meet?	○	○

This question is about seeing violence in the streets, in your neighborhood, or at school. Have you ever SEEN an act of violence towards some else, NOT BETWEEN MEMBERS OF YOU AND YOUR FAMILY, such as someone else being attacked, killed, shot at, beaten, or robbed?

- ◯ Yes
- ◯ No

This question in about violence that may have happened to YOU. This could have been while hanging out, while at school, or in the neighborhood. Have you ever EXPERIENCED an act of violence, NOT BY SOMEONE IN YOUR FAMILY, such as being attacked, shot at, stabbed, beaten, or robbed?

- ◯ Yes
- ◯ No

Sometimes kids see people in their family getting hurt, being beaten, punched, kicked, chocked, or thrown down hard by other family members (sometimes it is part of a fight). Have you ever SEEN this happen TO SOMEONE ELSE IN YOUR FAMILY?

- ◯ Yes
- ◯ No

Sometimes kids are hurt by people in their own family, such as being punched, kicked, chocked, or thrown down hard. HAVE YOU EVER EXPERIENCED BEING HURT BY SOMEONE IN YOUR FAMILY?

- ◯ Yes
- ◯ No

Have you ever deliberately inflicted harm on another person? Check all that apply

- ☐ Yes
- ☐ Yes, in self defence
- ☐ No
- ☐ Unsure
- ☐ Prefer not to answer

How did you deliberately inflict harm on another person? Please check all that apply.

- [ ] Beating up without the aid of a weapon (punching, kicking, choking, throwing down hard)
- [ ] Stabbing/knifing
- [ ] Shooting
- [ ] Other (please specify)

Have you ever been convicted of a crime?

- ◯ Yes
- ◯ No
- ◯ Unsure
- ◯ Prefer not to answer

Have you ever been in jail?

- ◯ Yes
- ◯ No
- ◯ Unsure
- ◯ Prefer not to answer

How many times have you been in jail?

- ◯ 1
- ◯ 2 - 5
- ◯ 6 - 9
- ◯ 10 - 13
- ◯ Other (please specify)

Did you have any kind of sexual intercourse while you were in jail?

- ◯ Yes
- ◯ No
- ◯ Unsure
- ◯ Prefer not to answer

Section G: Self Esteem

Below are listed a number of statements that reflect, in general, feelings of self worth and acceptance. Please read each statement carefully and indicate how much you agree or disagree that it applies to you.

	Strongly Agree	Agree	Disagree	Strongly Disagree
I feel that I'm a person of worth, at least on an equal basis with others.	◯	◯	◯	◯
I feel that I have a number of good qualities.	◯	◯	◯	◯
All in all, I am inclined to think I'm a failure.	◯	◯	◯	◯
I am able to do things as well as most other people.	◯	◯	◯	◯
I feel I do not have much to be proud of.	◯	◯	◯	◯
I like my body the way it is.	◯	◯	◯	◯
I take a positive attitude towards myself.	◯	◯	◯	◯
On the whole, I am satisfied with myself.	◯	◯	◯	◯
I wish I could have more respect for myself.	◯	◯	◯	◯
I certainly feel useless at times.	◯	◯	◯	◯
At times I think I'm no good at all.	◯	◯	◯	◯

Section H: Self-efficacy, Intentions and Beliefs

Below it a list of different situations regarding sexuality and condom use. For each question the answers can be No, Probably No, Probably Yes, and Yes.

	No	Probably No	Probably Yes	Yes	Not Applicable
Would you be able to avoid sex any time you didn't want it?	○	○	○	○	○
Would you be able to talk to your partner about his/her previous sexual activities?	○	○	○	○	○
Would you be able to use a condom every time you have sex?	○	○	○	○	○
Would you be able to use a condom during sex after you have been drinking or taking drugs?	○	○	○	○	○
For females, would you be able to refuse to have sex if your partner will not use a condom? For males, would you be able to refuse to have sex if your partner will not permit you to wear a condom?	○	○	○	○	○
Would you be able to talk about using condoms with your partner?	○	○	○	○	○

Below are a list of statements regarding condom use. Please read each statement carefully and indicate how much you agree or disagree with it.

	Strongly Agree	Agree	Neither Agree or Disagree	Disagree	Strongly Disagree
Asking to use a condom makes a partner think he or she is not trusted.	○	○	○	○	○
I would be embarrassed to use a condom.	○	○	○	○	○
Sex might be spoiled if my partner and I talked about using a condom.	○	○	○	○	○
It is hard to ask to use a condom if my partner and I are already using the pill.	○	○	○	○	○
My partner would be angry if I asked to use a condom.	○	○	○	○	○
It is hard to talk about ways not to get AIDS and other sexually transmitted diseases with a partner.	○	○	○	○	○
Condoms are a good way to help stop AIDS and other sexually transmitted diseases.	○	○	○	○	○
Condoms are safe to use.	○	○	○	○	○
Using condoms is the responsible thing to do.	○	○	○	○	○
By using condoms, I and my partner are less likely to get AIDS or other sexually transmitted diseases.	○	○	○	○	○
Using a condom shows you care about yourself and your partner.	○	○	○	○	○
If I used condoms, my partner would respect me.	○	○	○	○	○
Sex would be less exciting if condoms were used.	○	○	○	○	○
Sex is less romantic when using condoms.	○	○	○	○	○
Condoms make sex feel less good.	○	○	○	○	○

For each of the following statements, we would like you to tell us whether you agree or disagree.

	Agree	Disagree
It is more difficult to refuse sex with a partner who is older than you compared to a partner who is the same age as you.	◯	◯
Condom use is a shared responsibility for both partners.	◯	◯
It is cool to have a sexual partner who is older than you (5+ years).	◯	◯
It is okay to have sex with a sugar mommy, sugar daddy, or a person with whom you have sex so that they will buy you things.	◯	◯
Using condoms is a sign of not trusting your partner.	◯	◯
It is against my values for me to have sex while I am still a young person.	◯	◯
It is okay to pressure someone into have sex when they do not want to.	◯	◯
It is okay to have many sexual partners.	◯	◯
It is okay to have sex with my partner even though my partner does not want to.	◯	◯
It is okay to have sex when I do not want to but my partner insists on having sex.	◯	◯
It is okay for people my age to have sex.	◯	◯
In the future I want to be married or in a long-term relationship in which my partner and I only have sex with one another (a monogamous relationship).	◯	◯
Oral sex is not sex.	◯	◯
I have had dry sex, or I know people who have had dry sex. By dry sex we mean that before sexual intercourse, the vagina is dried with herbs or detergents.	◯	◯
Condoms carry viruses/diseases such as HIV/AIDS.	◯	◯
	◯	◯

Sex is pleasurable.	◯	◯
It is cool to have a boyfriend/girlfriend who is younger that you (5+ years).	◯	◯
It is okay to hit your boyfriend/girlfriend when you are angry with them.	◯	◯
It is okay to force your boyfriend/girlfriend to have sex when you are angry at them.	◯	◯
It is okay for my boyfriend/girlfriend to hit me when they are angry at me.	◯	◯
It is okay for my boyfriend/girlfriend to force me to have sex with them when they are angry at me.	◯	◯

For each of the following statements, we would like you to tell us whether you agree or disagree.

	Agree	Disagree
People who have AIDS are dirty.	○	○
People who have AIDS are cursed.	○	○
People who have AIDS should be ashamed.	○	○
It is safe for people who have AIDS to work with children.	○	○
People with AIDS must expect some restrictions on their freedom.	○	○
A person with AIDS must have done something wrong and deserves to be punished.	○	○
People who have AIDS should be isolated.	○	○
I do not want to be friends with someone who has AIDS.	○	○
People who have AIDS should not be allowed to work.	○	○

For each of the following statements, we would like you to tell us whether you agree or disagree.

	Agree	Disagree
Antiretroviral therapy is an effective treatment for HIV.	◯	◯
Antiretroviral therapy helps people with HIV live healthy, active lives.	◯	◯
Antiretroviral therapy reduces the amount of virus in an HIV-positive persons body preventing transmission of HIV.	◯	◯
Antiretroviral therapy can kill people.	◯	◯
Antiretroviral therapy is a scam by the pharmaceutical companies.	◯	◯
Antiretroviral therapy prevents pregnant women who have HIV from passing the infection on to their baby.	◯	◯
Antiretroviral therapy is needed to prevent HIV related deaths.	◯	◯
Antiretroviral therapy is needed to reduce HIV transmission in populations where many people are living with HIV.	◯	◯

Conclusion

One of Kganya Motsha's objectives is to reduce the fear surrounding HIV testing by making HIV testing a normal part of being a young person. We encourage and support all young people to come and test for HIV, even if they are not yet having sex. We will give you a coupon to come back to Kganya Motsha to have an HIV test as part of this study, and encourage you to do so, even if you are not yet having sexual intercourse.

Are you planning on returning to Kganya Motsha for an HIV test as part of this study?

- ◯ Yes
- ◯ No
- ◯ Unsure
- ◯ Prefer not to answer

Why are you planning on returning for an HIV test?

- ◯ I feel comfortable testing at Kganya Motsha
- ◯ I am curious to find out my HIV status
- ◯ Because I was asked as part of this survey/study
- ◯ I trust the staff at Kganya Motsha
- ◯ My mom, dad, or another relative died of HIV
- ◯ Prefer not to answer
- ◯ Other (please specify)

Why might you not return for an HIV test? Please check only the MAIN reason.

- ◯ Fear of being positive
- ◯ Fear of dying
- ◯ Fear of being rejected
- ◯ Fear that the results will be disclosed/my confidentiality violated
- ◯ Prefer not to answer
- ◯ Other (please specify)

Do you have a cell phone?

- ◯ Yes
- ◯ No

How did you get the cell phone?

- ◯ Parent
- ◯ Friend
- ◯ Bought it with pocket money
- ◯ Boyfriend/girlfriend
- ◯ Brother/sister
- ◯ Other (please specify)

What do you do with the cell phone? Please check all that apply.

- ☐ SMS with friends
- ☐ Call friends
- ☐ Use MXit
- ☐ Use Facebook
- ☐ Stay in touch with parents
- ☐ Work
- ☐ Play games
- ☐ Other (please specify)

Can you use someone else's cell phone?

- ◯ Yes
- ◯ No

Whose cell phone can you use? Please check all that apply.

- ☐ Parent
- ☐ Friend
- ☐ Boyfriend/girlfriend
- ☐ Brother/sister

Other (please specify)

How much time in a day do you spend using a cell phone?

- ◯ 0-1 hours
- ◯ 2-4 hours
- ◯ 5-7 hours
- ◯ 8-9 hours
- ◯ 10 or more hours

Do you have access to the internet?

- ◯ Yes
- ◯ No

How do you access the internet?

- ◯ Computer
- ◯ Cell phone

What do you use the internet for? Please check all that apply.

- ☐ MXit
- ☐ Facebook
- ☐ Homework
- ☐ Gaming
- ☐ Meeting others online
- ☐ Pornography
- ☐ Work
- ☐ Movies
- ☐ TV Shows
- ☐ Photography programs
- ☐ Shopping

Other (please specify)

____

How much do you use Facebook per day?

- ◯ 0-1 hours
- ◯ 2-4 hours
- ◯ 5-7 hours
- ◯ 8-9 hours
- ◯ 10 or more hours

Would you be interested in taking part in a study that uses websites like MXit and Facebook?

- ◯ Yes
- ◯ No

What is the MAIN reason that you would not want to take part in such a study?

How was the interview delivered to you?

- ◯ Interviewer administered
- ◯ Cell phone
- ◯ Computer

Did you like the way this interview was delivered?

- ◯ Yes
- ◯ No

Through what mode of interview delivery are youth like you more likely to provide the most honest answers?

- ◯ Interviewer administered
- ◯ Cell phone
- ◯ Computer

End time of the interview:

	HH		MM	AM/PM
Time:		:		
